# Susceptibility to e-cigarette adoption among tobacco-naïve youths: a cross-sectional study in Shenzhen, China

**DOI:** 10.3389/fpubh.2024.1320863

**Published:** 2024-05-16

**Authors:** Ruilin Yan, Yanhong Liu, Li Huang, Yanrou Li, Yun Huang, Jing Tong, Yongzheng Deng, Qing Yuan

**Affiliations:** ^1^Shenzhen Bao'an Center for Chronic Disease Control, Shenzhen, China; ^2^Bao'an Public Health Service Center of Shenzhen, Shenzhen, China

**Keywords:** e-cigarette, vaping, susceptibility, youth, smoking

## Abstract

**Background:**

The rise in e-cigarette use among youth is a significant global public health issue. It is important to identify those at increased risk and implement effective strategies to reduce e-cigarette popularity among the youth.

**Objective:**

This study aims to identify predictors of e-cigarette uptake in youths with no prior tobacco use, considering individual, familial and the broader societal environmental factors.

**Methods:**

For this investigation, a group of 2,487 tobacco-free youths was selected from 15 high schools in Shenzhen, China. Susceptibility to e-cigarettes was determined by assessing the possibility of future use and the openness to trying e-cigarettes if presented by friends. Both chi-square tests and logistic regression were applied to identify factors linked to susceptibility to e-cigarette use.

**Results:**

Among the respondents, 5.5% (*n* = 136) were found to be susceptible to e-cigarette use. The analysis revealed factors tied to this risk: perceptions of e-cigarettes, the impact of vaping peers, paternal parenting styles, the extent of social support, exposure to messages both for and against e-cigarettes use, and secondhand smoke (SHS) exposure. Youths who downplayed the addictive nature of e-cigarettes (aOR = 2.01; 95% CI: 1.14–3.55; *p* = 0.016), those with friends who engaged in vaping (aOR = 3.43–7.64; 95%CI: 2.36–20.42; *p* < 0.001), those experiencing over-protective or rejective maternal parenting (aOR = 1.68–3.01; 95%CI: 1.11–5.77; *p* = 0.001–0.014) or rejective paternal parenting (aOR = 3.63; 95%CI: 1.99–6.59; *p* < 0.001), those aware of e-cigarette advertisements (aOR = 1.82; 95%CI: 1.28–2.60; *p* = 0.001), and those exposed to SHS at home (aOR = 1.68; 95%CI: 1.17–2.41; *p* = 0.005) or at public places (aOR = 1.72–1.79; 95%CI: 1.21–2.57; *p* = 0.002–0.003) were more prone to e-cigarettes. In contrast, youths who believed using e-cigarettes reduces one’s attractiveness (aOR = 0.34; 95%CI: 0.16–0.72; *p* = 0.005) or perceived that vaping made social interactions less enjoyable (aOR = 0.26; 95%CI: 0.12–0.58; *p* = 0.001), those who benefited from high social support (aOR = 0.30–0.60; 95%CI: 0.17–0.97; *p* < 0.001), and those who noticed message about e-cigarettes’ adverse consequence (aOR = 0.54; 95%CI: 0.38–0.77; *p* = 0.001) were less likely to be inclined toward e-cigarette use.

**Conclusion:**

The propensity of the youth to e-cigarette usage is shaped by a multiple element. An all-encompassing strategy that addresses the individual, familial, and the broader societal aspects is imperative for the effective prevention of e-cigarette initiation among youth.

## 1 Introduction

The use of e-cigarettes has become an issue of global health concern, with its appeal spreading rapidly, especially among the younger demographic. In the United States, from 2011 to 2020, there was a substantial climb in e-cigarette usage among high schoolers within the past month, jumping from 1.5 to 19.6%. This represented around 3.02 million high school and 550,000 middle school students actively using e-cigarettes (1, 2). Due to this surge, the U.S. Food and Drug Administration labeled the situation as an “epidemic” among the youth (3). Canada and various European nations have also reported similar surges in youth e-cigarette use (4, 5). In China, the birthplace and main manufacturer of e-cigarettes, there has been a noticeable drop in traditional cigarette smoking among young people from 2014 to 2021, while e-cigarette usage has seen a threefold increase (6). Internationally, e-cigarettes have overtaken conventional cigarettes as the foremost tobacco product among the youth (1–7).

The negative health impacts of e-cigarette use have been well-established in the existing literature. E-cigarettes carry numerous toxic chemicals also present in traditional cigarettes, potentially leading to heart and lung diseases (8, 9). The flavoring chemicals in e-cigarettes can turn into carcinogens when heated (10). Moreover, e-cigarettes generally contain nicotine, which is highly addictive and can negatively impact brain development over the long term (11, 12), possibly leading to addictions to tobacco and other substances (13–15). With traditional cigarette smoking rates in decline in the youth, the rise of e-cigarette consumption threatens to halt or reverse this trend. The public health sector is particularly worried about young individuals who have not previously smoked, prompting a call for immediate measures to safeguard them from the dangers of e-cigarettes.

Recognizing and preventing at-risk youths from starting to use e-cigarettes is of great importance due to their prevalent use and health risks. Susceptibility to e-cigarette is understood as the absence of a firm decision not to use them (16). Studies have indicated that susceptibility is a key indicator of future e-cigarette use (17–19). For example, research by Bold et al. revealed that susceptible youths were over five times more likely to become regular e-cigarette users within 6 months than those who were not susceptible (15). Identifying these vulnerable young people is crucial for crafting successful preventative strategies.

There are numerous surveys on the current situation of adolescent vaping of e-cigarettes and related risk factors. However, there is less attention on the susceptibility to e-cigarette use among youths who had no prior experience with smoking traditional or electronic cigarettes, except for some studies conducted in North America, Latin America, Australia, and Spain (20–26). The ecological model of health behavior suggests that the occurrence and development of health behaviors, as tobacco and alcohol abuse, are influenced by multiple factors at individual, familial, and the broader societal level (27). Yet, comprehensive and in-depth investigations into these influences to e-cigarette use susceptibility among tobacco-naïve youths are uncommon. For instance, Tackett (21), Copp (22), and Williams (24) examined the susceptibility of American and Canadian adolescents to using e-cigarettes, mainly analyzing the influence from individual factors such as demographic information as well as perceptions of tobacco use and substance use. Gottschlich (23) conducted a study on the susceptibility of youth to e-cigarettes in Guatemala, focusing mainly on demographic factors, personal substance abuse, and the influence of parental and peer tobacco use behaviors. Carey (25) and Kwon (26) examined factors from individual, family, and societal environment as predictors of youth e-cigarette use susceptibility, but did not include social support and parenting style, despite their proven connection to traditional tobacco usage (28–31). To fill in the research gap, we conduct the present study and seek to explore factors associated with e-cigarette use susceptibility among tobacco-naïve youths, including demographic characteristics, perceptions of e-cigarettes, parents and peers’ behavior, pro-and anti-e-cigarettes messaging exposure, and secondhand smoke (SHS) exposure from individual, familial, and the broader societal level.

## 2 Materials and methods

### 2.1 Participants and procedure

In collaboration with the Bao’an District Education Office, our team executed a sectional study within schools, targeting 12 to 17-year-old middle and high school students in Shenzhen’s Bao’an District, over the period from October to December 2021. To select a representative group of students, a multi-stage stratified cluster random sampling method was employed. Initially, a random draw was performed to pick eight middle schools, five general high schools, and two vocational high schools, based on school category. All 15 schools selected agreed to participate. Subsequently, using a random selection process, we chose one or two classrooms from each grade within these schools, and all students from these classrooms were asked to join the study.

The initial sample size was determined by anticipating a 7.0% susceptibility to e-cigarette use among tobacco-naïve youths (20), with a 0.05 alpha level and a 17% margin of error, which suggested the need for 1766 participants. To account for the possibility of non-response and incomplete data typically associated with cluster sampling, the sample size was augmented by 30%, bringing the total to 2,296 participants. Considering an estimated 20% prevalence of tobacco use history among the student population (6), it was decided to survey 2,870 students, rounding up to 2,900 participants for the final sample.

The study’s approach received approval from the Ethics Review Committee at the Shenzhen Bao’an Center for Chronic Disease Control. Both students and their guardians were fully informed of the study’s purpose and details and were offered the option to participate on a voluntary basis. The students were asked to fill out structured questionnaires in their classrooms, in the absence of their teachers but under the supervision of a trained researcher, to maintain the study’s integrity. Ensuring privacy, students provided their responses anonymously, the questionnaires were collected immediately, and the data was kept confidential, being used solely for the study. It was made clear to the participants that they had the liberty to skip any question that made them feel uneasy.

### 2.2 Instrument

The investigation employed the Chinese Youth Tobacco Survey questionnaire, which was designed by the Chinese Center for Disease Control and Prevention. This tool is grounded in the foundational questionnaire of the Global Youth Tobacco Survey, a widely acknowledged benchmark for the consistent tracking of tobacco consumption among youths on a global scale. It incorporates additional queries that cater to the unique context of China. Moreover, the Simplified Parenting Style Scale (CSPSS) and the Multidimensional Scale of Perceived Social Support (MSPSS) measuring parenting style and social support were applied. Data were gathered concerning the primary outcome of susceptibility to e-cigarette, alongside exposure factors spanning demographic characteristics, perceptions of e-cigarettes, parents and peers’ behavior, and the broader societal environmental influence. An elaboration of these factors is provided.

#### 2.2.1 Susceptibility to e-cigarette use

Drawing from the framework of the Global Youth Tobacco Survey and prior studies (17, 19, 32), the likelihood of engaging in e-cigarette use was evaluated with two questions: “Do you think that you will use an e-cigarette in the next 12 months?” and “If one of your close friends offered you an e-cigarette, would you use it?” The options for responses included “definitely not,” “probably not,” “probably yes,” or “definitely yes.” Respondents selected the choice most representative of their stance. Those who responded “definitely not” to both questions were categorized as non-susceptible, while any other combination of answers indicated susceptibility.

#### 2.2.2 Demographic characteristics

Attributes such as age, sex, grade, type of school, amount of pocket money, family structure, and father’s and mother’s education level were factored in.

#### 2.2.3 Perceptions of e-cigarette

Youth perceptions of e-cigarettes were gaged through three specific questions--"Do you think it is easy to be addicted once someone starts vaping?” “Do you think vaping makes people seem more attractive?” and “Do you think vaping helps people feel more comfortable in social gatherings?”

#### 2.2.4 Parents and peers’ behavior

Parents smoking, friends vaping, parenting style, and social support from family, friends, and significant others were factored in.

Parenting styles were evaluated using the Chinese form of the Simplified Parenting Style Scale (CSPSS) (33), which consists of 21 questions and measures the perceived parenting styles by the youth, covering three aspects: emotional support, punitive behavior, and excessive protectiveness. Survey items were scored on a 4-point scale from 1 (“never”) to 4 (“always”), with participants being assigned to the parenting style they rated most highly. The scale demonstrated strong reliability, with a Cronbach’s alpha of 0.88.

Social support was evaluated using the Multidimensional Scale of Perceived Social Support (MSPSS) by Zimet and colleagues, which consists of 12 items and evaluates support from family, friends, and significant others. Responses were on a scale from 1 to 7, with total scores ranging from 12 to 84. The MSPSS exhibited strong reliability, with a Cronbach’s alpha of 0.96.

#### 2.2.5 The broader societal environmental influence

The study also looked at the broader societal environmental influences, including pro-and anti-e-cigarette messaging exposure, and SHS exposure. Pro-and anti-e-cigarette messaging exposure was measured with two questions: “Have you ever noticed e-cigarette advertising in the past 30 days?” and “Have you ever noticed message about the negative effects of e-cigarette use in the past 30 days?” SHS exposure at home, at indoor and outdoor public places, and in public transportation during the past 7 days was measured.

### 2.3 Statistical analysis

The analysis was conducted using the SPSS Statistics software, version 22.0. Categorical variables were summarized using frequencies and percentages, while continuous variables were presented as means with standard deviations. Chi-square tests of independence were performed to detect disparities in susceptibility among different groups of youths. Bivariate logistic regression and multivariable logistic regression was employed to calculate unadjusted odds ratio (OR) and adjusted odd ratios (aOR) and their 95% confidence intervals (CI) for the factor under consideration. Exposure factors spanning perceptions of e-cigarettes, parents and peers’ behavior, and the broader societal environmental influence were separately brought into the bivariate logistic regression and multivariable logistic regression, regardless of their statistical significance in the chi-square tests. In the multivariable logistic regression, demographic factors that were statistically significantly associated with susceptibility in the chi-square tests were adjusted for as covariates. A trend analysis was incorporated within the chi-square tests and the regression model to evaluate whether a relationship trend exists between factors like age, the amount of pocket money, and social support scores, and the propensity for using e-cigarettes. Linear trends were examined by using age as a continuous variable (since the participants’ ages were primarily concentrated in the narrow range of 12 to 17 years old, no categorization by age was conducted), as well as using the median values of the amount of pocket money quartiles and social support score quartiles as continuous variables in the logistic regression. The threshold for statistical significance was established at *p* < 0.05 for all two-tailed tests.

## 3 Results

### 3.1 Sample characteristics

In total, we approached 2,929 students from 15 schools to partake in our research, from which 2,915 (99.5%) successfully filled out the surveys. A subset of 2,487 individuals (85.3%) indicated they had no prior experience with smoking traditional or electronic cigarettes, and their data was subsequently used in our definitive evaluation. The average age of participants was 14.3 years (SD: 1.7, Rang: 12–17). Data inclusive of demographic characteristics, perceptions of e-cigarettes, parents and peers’ behavior, and the broader societal environmental factors, are detailed in Table 1.

**Table 1:** Characteristics of tobacco-naïve youths (*N* = 2,487).

Variable		n	Weighted (%)
Demographic characteristics			
Sex	Male	1,393	56.0
	Female	1,094	44.0
Age (years)	12	389	15.6
	13	605	24.3
	14	501	20.1
	15	367	14.8
	16	301	12.1
	17	324	13.0
Type of school	Middle school	1,630	65.5
	General high school	583	23.4
	Vocational high school	274	11.0
Amount of pocket money (CNY/week)^a^	Quartile 1	877	35.6
	Quartile 2	397	16.1
	Quartile 3	728	29.5
	Quartile 4	464	18.8
Family structure	Complete family	2,221	89.3
	Incomplete family	266	10.7
Education level of the mother	Junior school or below	821	33.0
	High school	683	27.5
	College or above	720	29.0
	Unknown	263	10.6
Education level of the father	Junior school or below	698	28.1
	High school	705	28.3
	College or above	834	33.5
	Unknown	250	10.1
Perceptions of e-cigarettes			
Do you think it is easy to become addicted once someone starts vaping?	Maybe easy	2080	83.6
	Maybe not easy	150	6.0
	Unknown	257	10.3
Do you think vaping makes people appear more attractive?	More attractive	86	3.5
	Less attractive	1818	73.1
	No difference	583	23.4
Do you think vaping helps people feel more comfortable in social gatherings?	More comfortable	55	2.2
	More uncomfortable	2,223	89.4
	No difference	209	8.4
Parents and peers’ behavior			
Parents smoking	Both being nonsmoker	1,408	56.6
	Father or mother being a smoker	1,079	43.4
Friends vaping	No one	1809	72.7
	Someone	652	26.2
	Most/All	26	1.0
Parenting style of the mother	Emotional warmth	1917	77.5
	Over-protecting	453	18.3
	Rejecting	105	4.2
Parenting style of the father	Emotional warmth	1894	76.6
	Over-protective	466	18.9
	Rejective	111	4.5
Social support score^a^	Quartile 1	645	26.1
	Quartile 2	632	25.5
	Quartile 3	603	24.4
	Quartile 4	596	24.1
The broader societal environmental factors			
Have you noticed any e-cigarette advertising in the past 30 days?	No	1,686	67.8
	Yes	801	32.2
Have you noticed any message about the negative effects of vaping in the past 30 days?	No	1,144	46.0
	Yes	1,343	54.0
Exposed to SHS at home in the past 7 days	Yes	724	29.1
	No	1763	70.9
Exposed to SHS at indoor public places in the past 7 days	No	1,144	46.0
	Yes	1,087	43.7
Exposed to SHS at outdoor public places in the past 7 days	No	1,209	48.6
	Yes	1,278	51.4
Exposed to SHS in public transportation in the past 7 days	No	1,209	48.6
	Yes	1,316	52.9

SHS, secondhand smoke. ^a^Grouping by Quartiles.

### 3.2 Characteristics of youth susceptible to e-cigarette use

Among the 2,487 students, 136 individuals (5.5%) were identified as being susceptible to trying e-cigarettes. As depicted in Table 2, there were notable differences in a multitude of factors between youths susceptible to e-cigarette use and their non-susceptible counterparts, especially in perceptions of e-cigarettes, influence of peers who vape, parenting styles, levels of social support, pro-and anti-e-cigarette information exposure, and SHS exposure.

**Table 2:** Susceptibility to e-cigarette use by characteristics (*n* = 2,487).

Variable		Susceptible to e-cigarette use		*p*
		No n(%)	Yes n(%)	
Demographic characteristics				
Sex	Male	1,310 (94.0)	83 (6.0)	0.225
	Female	1,041 (95.2)	53 (4.8)	
Age (years)	12	373 (95.9)	16 (4.1)	0.002^*^
	13	578 (95.5)	27 (4.5)	
	14	479 (95.6)	22 (4.4)	
	15	344 (93.7)	23 (6.3)	
	16	281 (93.4)	20 (6.6)	
	17	296 (91.4)	28 (8.6)	
Type of school	Middle school	1,554 (95.3)	76 (4.7)	0.009
	General high school	548 (94.0)	35 (6.0)	
	Vocational high school	249 (90.9)	25 (9.1)	
Amount of pocket money (CNY/week)^a^	Quartile 1	836 (95.3)	41 (4.7)	0.044^*^
	Quartile 2	376 (94.7)	21 (5.3)	
	Quartile 3	690 (94.8)	38 (5.2)	
	Quartile 4	428 (92.2)	36 (7.8)	
Family structure	Complete family	2,103 (94.7)	118 (5.3)	0.324
	Incomplete family	248 (93.2)	18 (6.8)	
Education level of the mother	Junior school or below	774 (94.3)	47 (5.7)	0.412
	High school	642 (94.0)	41 (6.0)	
	College or above	689 (95.7)	31 (4.3)	
	Unknown	246 (93.5)	17 (6.5)	
Education level of the father	Junior school or below	651 (93.3)	47 (6.7)	0.094
	High school	669 (94.9)	36 (5.1)	
	College or above	799 (95.8)	35 (4.2)	
	Unknown	232 (92.8)	18 (7.2)	
Perceptions of e-cigarettes				
Do you think it is easy to become addicted once someone starts vaping?	Maybe easy	1968 (94.6)	112 (5.4)	0.005
	Maybe not easy	134 (89.3)	16 (10.7)	
	Unknown	249 (96.9)	8 (3.1)	
Do you think vaping makes people appear more attractive?	More attractive	77 (89.5)	9 (10.5)	<0.001
	Less attractive	1743 (95.9)	75 (4.1)	
	No difference	531 (91.1)	52 (8.9)	
Do you think vaping helps people feel more comfortable in social gatherings?	More comfortable	47 (85.5)	8 (14.5)	<0.001
	Less comfortable	2,118 (95.3)	105 (4.7)	
	No difference	186 (89.0)	23 (11.0)	
Parents and peers’ behavior				
Parents smoking	Both being nonsmoker	1,342 (95.3)	66 (4.7)	0.050
	Father or mother being a smoker	1,009 (93.5)	70 (6.5)	
Friends vaping	No one	1750 (96.7)	59 (3.3)	<0.001
	Someone	581 (89.1)	71 (10.9)	
	Most/All	20 (76.9)	6 (23.1)	
Parenting style of the mother	Emotional warmth	1829 (95.4)	88 (4.6)	0.001
	Over-protective	418 (92.3)	35 (7.7)	
	Rejective	93 (88.6)	12 (11.4)	
Parenting style of the father	Emotional warmth	1807 (95.4)	87 (4.6)	<0.001
	Over-protective	434 (93.1)	32 (6.9)	
	Rejective	96 (86.5)	15 (13.5)	
Social support score^a^	Quartile 1	594 (92.1)	51 (7.9)	<0.001^*^
	Quartile 2	590 (93.4)	42 (6.6)	
	Quartile 3	575 (95.4)	28 (4.6)	
	Quartile 4	582 (97.7)	14 (2.3)	
The broader societal environmental factors				
Have you noticed any e-cigarette advertising in the past 30 days?	No	1,613 (95.7)	73 (4.3)	<0.001
	Yes	738 (92.1)	63 (7.9)	
Have you noticed any message about the negative effects of vaping in the past 30 days?	No	1,062 (92.8)	82 (7.2)	0.001
	Yes	1,289 (96.0)	54 (4.0)	
Exposed to SHS at home in the past 7 days.	No	1,681 (95.3)	82 (4.7)	0.005
	Yes	670 (92.5)	54 (7.5)	
Exposed to SHS at indoor public places in the past 7 days	No	1,314 (95.8)	59 (4.2)	0.002
	Yes	1,010 (92.9)	77 (7.1)	
Exposed to SHS at outdoor public places in the past 7 days	No	1,160 (95.9)	49 (4.1)	0.003
	Yes	1,191 (93.2)	87 (6.8)	
Exposed to SHS in public transportation in the past 7 days	No	1,109 (94.7)	62 (5.3)	0.719
	Yes	1,242 (94.4)	74 (5.6)	

SHS, secondhand smoke.^a^Grouping by Quartiles.^*^p for trend.

### 3.3 Factors associated with susceptibility to e-cigarette use

Table 3 presents the outcomes of logistic regression analysis, which identified a range of factors significantly associated with an increased risk of susceptibility to e-cigarette usage. These factors include perceptions of e-cigarettes, the impact of friends who vape, parenting styles, levels of social support, SHS exposure, and pro-and anti-e-cigarette information exposure. Youths who perceived e-cigarettes to be less addictive (aOR = 2.01; 95% CI: 1.14–3.55; *p* = 0.016), those with friends who engaged in vaping (aOR = 3.43–7.64; 95%CI: 2.36–20.42; *p* < 0.001), those subject to an over-protective or rejective parenting approach from their mothers (aOR = 1.68–3.01; 95%CI: 1.11–5.77; *p* = 0.001–0.014) or a rejective parenting approach from their fathers (aOR = 3.63; 95%CI: 1.99–6.59; *p* < 0.001), those aware of e-cigarette advertisements (aOR = 1.82; 95%CI: 1.28–2.60; *p* = 0.001), and those exposed to SHS at home (aOR = 1.68; 95%CI: 1.17–2.41; *p* = 0.005) or at public places (aOR = 1.72–1.79; 95%CI: 1.21–2.57; *p* = 0.002–0.003) were all more prone to considering e-cigarette use. In contrast, youths who believed using e-cigarettes reduces one’s attractiveness (aOR = 0.34; 95%CI: 0.16–0.72; p = 0.005) or believed that vaping made social interactions less enjoyable (aOR = 0.26; 95%CI: 0.12–0.58; *p* = 0.001), those who benefited from high social support (aOR = 0.30–0.60; 95%CI: 0.17–0.97; *p* < 0.001), and those who noticed message about e-cigarettes’ adverse consequence (aOR = 0.54; 95%CI: 0.38–0.77; *p* = 0.001) were less likely to be inclined toward e-cigarette use.

**Table 3:** Factors associated with susceptibility to e-cigarette use: bivariate and multivariable logistic regression analyses (*n* = 2,487).

Variable		Univariate analysis		multivariable analysis	
		OR(95%CI)	*p*	aOR(95%CI)^a^	*p*
Perceptions of e-cigarettes					
Do you think it is easy to become addicted once someone starts vaping?	Maybe easy	Reference		Reference	
	Maybe not easy	2.10 (1.21–3.65)	0.009	2.01 (1.14–3.55)	0.016
	Unknown	0.57 (0.27–1.17)	0.124	0.54 (0.26–1.13)	0.103
Do you think vaping makes people appear more attractive?	More attractive	Reference		Reference	
	Less attractive	0.37 (0.18–0.76)	0.007	0.34 (0.16–0.72)	0.005
	No difference	0.84 (0.40–1.77)	0.642	0.74 (0.35–1.59)	0.440
Do you think vaping helps people feel more comfortable in social gatherings?	More comfortable	Reference		Reference	
	Less comfortable	0.29 (0.13–0.63)	0.002	0.26 (0.12–0.58)	0.001
	No difference	0.73 (0.31–1.73)	0.469	0.59 (0.24–1.42)	0.236
Parents and peers’ behavior					
Parents smoking	Both being nonsmoker	Reference		Reference	
	Father or mother being a smoker	1.65 (1.16–2.36)	0.006	1.34 (0.94–1.91)	0.102
Friends vaping	No one	Reference		Reference	
	Someone	3.63 (2.53–5.18)	<0.001	3.43 (2.36–4.98)	<0.001
	Most/All	8.90 (3.45–22.97)	<0.001	7.64 (2.86–20.42)	<0.001
Parenting style of the mother	Emotional warmth	Reference		Reference	
	Over-protective	1.74 (1.16–2.61)	0.007	1.68 (1.11–2.53)	0.014
	Rejective	2.68 (1.42–5.08)	0.002	3.01 (1.57–5.77)	0.001
Parenting style of the father	Emotional warmth	Reference		Reference	
	Over-protective	1.53 (1.01–2.33)	0.046	1.48 (0.97–2.26)	0.073
	Rejective	3.25 (1.81–5.83)	<0.001	3.63 (1.99–6.59)	<0.001
Social support score	Quartile 1	Reference	<0.001^*^	Reference	
	Quartile 2	0.83 (0.54–1.27)		0.86 (0.56–1.32)	<0.001^*^
	Quartile 3	0.57 (0.35–0.91)		0.60 (0.37–0.97)	
	Quartile 4	0.28 (0.15–0.51)		0.30 (0.17–0.56)	
The broader societal environmental factors					
Have you noticed any e-cigarette advertising in the past 30 days?	No	Reference		Reference	
	Yes	1.89 (1.33–2.67)	<0.001	1.82 (1.28–2.60)	0.001
Have you noticed any message about the negative effects of vaping in the past 30 days?	No	Reference		Reference	
	Yes	0.54 (0.38–0.77)	0.001	0.54 (0.38–0.77)	0.001
Exposed to SHS at home in the past 7 days.	No	Reference		Reference	
	Yes	1.65 (1.16–2.36)	0.006	1.68 (1.17–2.41)	0.005
Exposed to SHS at indoor public places in the past 7 days	No	Reference		Reference	
	Yes	1.73 (1.22–2.46)	0.002	1.72 (1.21–2.44)	0.003
Exposed to SHS at outdoor public places in the past 7 days	No	Reference		Reference	
	Yes	1.73 (1.21–2.48)	0.003	1.79 (1.24–2.57)	0.002
Exposed to SHS in public transportation in the past 7 days	No	Reference		Reference	
	Yes	1.07 (0.75–1.51)	0.719	1.01 (0.71–1.45)	0.948

SHS, secondhand smoke; OR, odds ratio; aOR, adjusted odds ratio.^a^Adjusted for demographic characteristics including age (by using age as a continuous variable), type of school, amount of pocket money (by using medians of quartiles as a continuous variable).^*^p for trend. Linear trend was examined by using medians of social support score quartiles as a continuous variable.

## 4 Discussion

### 4.1 Main findings

This research serves as one of the preliminary explorations into the detailed analysis of various elements that contribute to the susceptibility of youths without prior tobacco exposure to the enticement of e-cigarette use. Our results show that 5.5% of these youths in Shenzhen are susceptible to e-cigarette use, a statistic that is comparable to the 7.67% observed in Shanghai (20) and considerably less than the 24.2% reported in the United States (25). Additionally, our research identifies a range of factors at individual, familial, and broader societal levels that affect this willingness to engage in e-cigarette usage, which we will detail further in our discussion.

### 4.2 Perception of e-cigarette

The study reveals that youth impressions of e-cigarette usage significantly shape their openness to trying them, corroborating previous research (34, 35). Youths who are under the impression that e-cigarette addiction is not a likely outcome are over twice as prone to consider using e-cigarettes compared to those who acknowledge the ease of addiction. On the flip side, the belief that e-cigarette usage detracts from social enjoyment and one’s appeal can diminish the likelihood of susceptibility by 60%. It is vital for the prevention of e-cigarette initiation among tobacco-naïve youths and for encouraging tobacco users to quit that they possess accurate information regarding the addictive and detrimental nature of e-cigarettes (34, 35). Our results underscore the importance of incorporating educational efforts about the dangers of e-cigarettes and fostering correct perceptions among youths into prevention and control policies.

### 4.3 Parents and peers’ behavior

For the initial time, our study investigated the link between parenting styles and the predisposition of youths to e-cigarette use, revealing that those who had parents with an over-protective or rejective parenting style are more inclined to be open to e-cigarettes than those who had parents with an emotional warmth parenting style. This aligns with earlier research highlighting a similar connection between parenting styles and youth smoking (27). Drawn from the stress-coping framework, parenting marked by emotional warmth is shown to bolster young people’s resistance to stress and reduce their turn toward e-cigarettes (36). The emotional support from parents can provide a sense of security and a safeguard against the negative impacts of life’s stressors on youth e-cigarette use. Conversely, overprotectiveness by parents may be perceived by adolescents as a form of external intrusion, and a high level of rejection could become a source of negative stimuli. Both factors could trigger adverse emotions and elevate the susceptibility of youths to e-cigarette use. Moreover, youths often view their parents as role models, and the absence of parental warmth can lead to poor peer relationships and a higher risk of e-cigarette allure. Our findings emphasize the critical role of parents in adopting a supportive parenting approach to aid youths in avoiding e-cigarette use.

It was observed that a strong perception of social support inversely affects the likelihood of adolescents’ susceptibility to e-cigarettes, a finding that echoes research on traditional cigarette consumption (37–39). Steptoe’s research on the interplay between stress perception, social support, and health behaviors revealed a noticeable uptick in traditional cigarette smoking during stressful times, particularly among students with insufficient social support (37). Similar patterns have been noted among commercial truck drivers (38) and adolescents (39), with those experiencing lower levels of social support showing a higher propensity to smoke. Managing stress, anxiety, boredom, or loneliness is one of important motivators for tobacco use among the youth. The buffering role of social support against the influence of adverse emotions on hazardous behaviors like e-cigarette use is well-documented (40). These insights are pivotal for guiding future prevention initiatives aimed at bolstering social support for youths, enabling them to confront life’s challenges in a positive manner and steer clear of e-cigarettes.

The study also confirms that having friends who use e-cigarettes raises the probability of a young person being open to trying them, due to perceived social acceptability and accessibility via their social circle, which aligns with existing research (41, 42). This suggests the importance of peer education in aiding current users to quit and bolstering non-users’ resilience against peer influence.

### 4.4 Broader societal environmental influence

The likelihood of youths taking up the use of e-cigarettes is heavily influenced by their exposure to messages that endorse e-cigarettes. Media exposure plays a significant role in fueling youth interest in e-cigarettes by glamorizing e-cigarettes, minimizing the risks of addiction and harm, and fostering positive beliefs and expectations (43, 44). Consequently, it is of utmost importance to intensify the regulatory scrutiny on the marketing practices of e-cigarettes, particularly those that appeal to the younger population. Steps to achieve this could include prohibiting advertisements that make misleading or vague health claims and mandating that e-cigarette packaging prominently displays health warnings. Research indicates that a 10% price increase of e-cigarettes results in an 8.2% decline in their sales, pointing to the effectiveness of strategies like taxation and price control as tools to curb e-cigarette use among young people (45).

Consistent with prior investigations (20, 26, 46–48), research corroborates that SHS exposure is associated with an elevated likelihood of youth experimenting with e-cigarettes. This exposure could act as a gateway from not smoking to smoking, possibly due to repeated exposure to nicotine fostering tolerance and subsequent changes in behavior related to nicotine addiction (49). Human development theories further illustrate the significant role other social members play in shaping youth behavior, as they often emulate the smoking behavior observed. Our study emphasizes the importance of maintaining smoke-free environments to deter young people from starting to use e-cigarettes. In 2014, the “Shenzhen Special Economic Zone Smoking Control Ordinance” was enacted, prohibiting smoking and vaping in public areas. Despite this, our research indicates that in the home environment, approximately 30% of adolescents are affected by secondhand smoke, while in public spaces this figure exceeds 40%, a proportion significantly higher than the 15.5% reported in the United States (50). This situation clearly reveals the widespread and profound impact of secondhand smoke on young people, highlighting the urgency of strengthening tobacco control measures and enforcing relevant regulations, with the aim of creating a smoke-free living environment.

### 4.5 Limitation

This research has its constraints, notably a participant pool drawn solely from the urban area of Shenzhen, which may not reflect the situation in other areas or nations. It is therefore suggested that subsequent studies incorporate a broader demographic. The study’s cross-sectional nature also means it cannot establish cause and effect in the relationship between susceptibility and the identified elements. To gain a deeper understanding of these determinants, future studies should be longitudinal. Moreover, while numerous factors were taken into account, it is possible that certain variables or confounding factors were missed. Future investigations should endeavor to include these overlooked aspects.

## 5 Conclusion

Our research has identified a range of elements at individual, familial, and the broad societal level that contribute to the likelihood of tobacco-naïve youths starting to use e-cigarettes. The evidence we have gathered indicates that a comprehensive strategy that encompasses the individual, family unit, broader community, and regulatory policies is essential to deter the commencement of e-cigarette usage among the youth.

## Data availability statement

The raw data supporting the conclusions of this article will be made available by the authors, without undue reservation.

## Ethics statement

The studies involving humans were approved by Ethics Review Committee of Shenzhen Bao’an Center for Chronic Disease Control. The studies were conducted in accordance with the local legislation and institutional requirements. Written informed consent for participation in this study was provided by the participants’ legal guardians/next of kin.

## Author contributions

RY: Conceptualization, Writing – original draft, Investigation, Methodology. YLiu: Investigation, Methodology, Writing – original draft. LH: Investigation, Methodology, Writing – original draft. YLi: Investigation, Methodology, Writing – original draft. YH: Investigation, Methodology, Writing – original draft. JT: Writing – review & editing. YD: Methodology, Project administration, Writing – review & editing. QY: Project administration, Writing – review & editing.
